# Supplementary material for: Evaluation of haplotype-aware long-read error correction with hifieval
Source: Bioinformatics. 2023 Oct 18;39(10):btad631. doi: 10.1093/bioinformatics/btad631 (PMC10612404; doi:10.1093/bioinformatics/btad631)
Supplement: btad631_Supplementary_Data [file btad631_supplementary_data.pdf]

Hifieval Supplementary Material

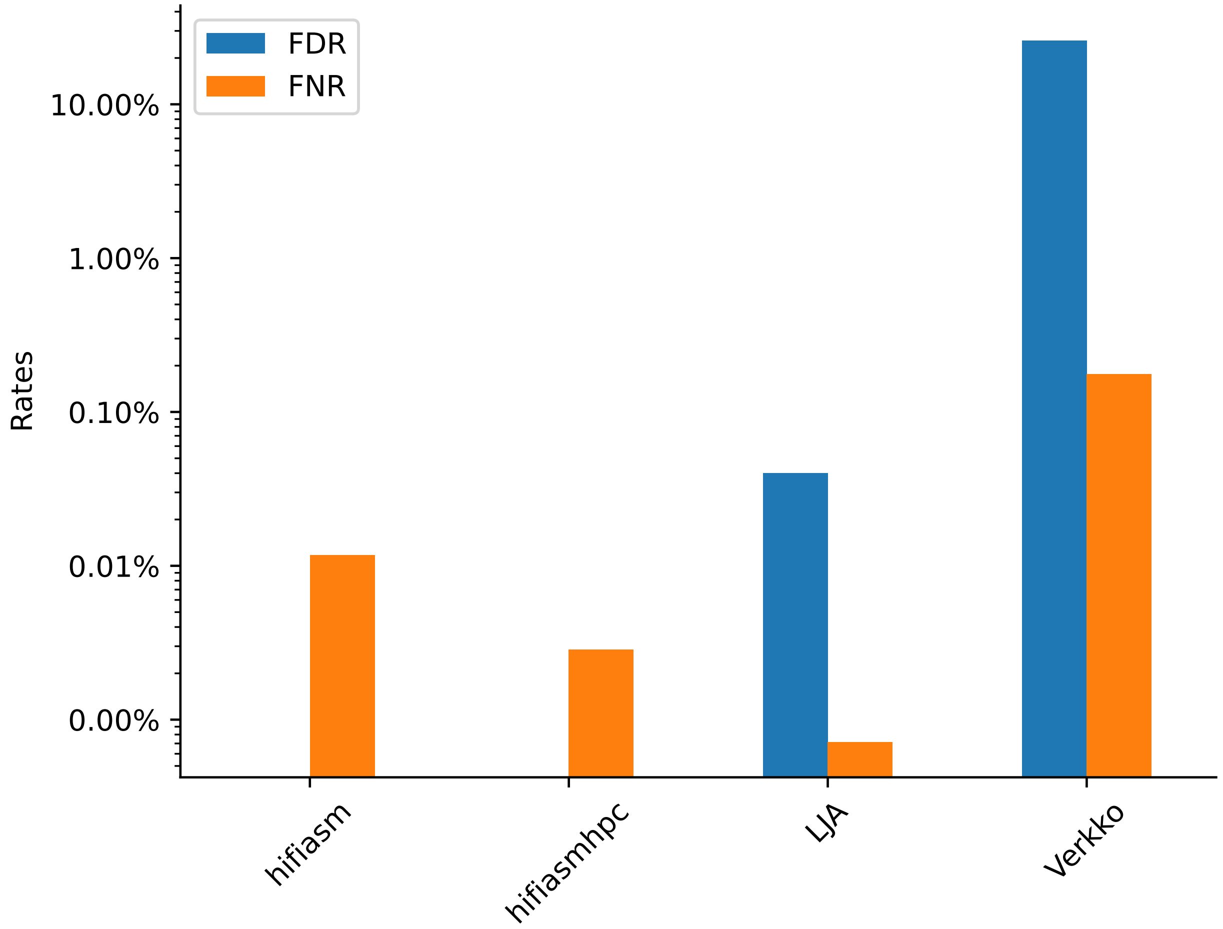

Supplementary Figure 1: The FNR and FDR of Reads Error Correction of each tool on *E. coli*

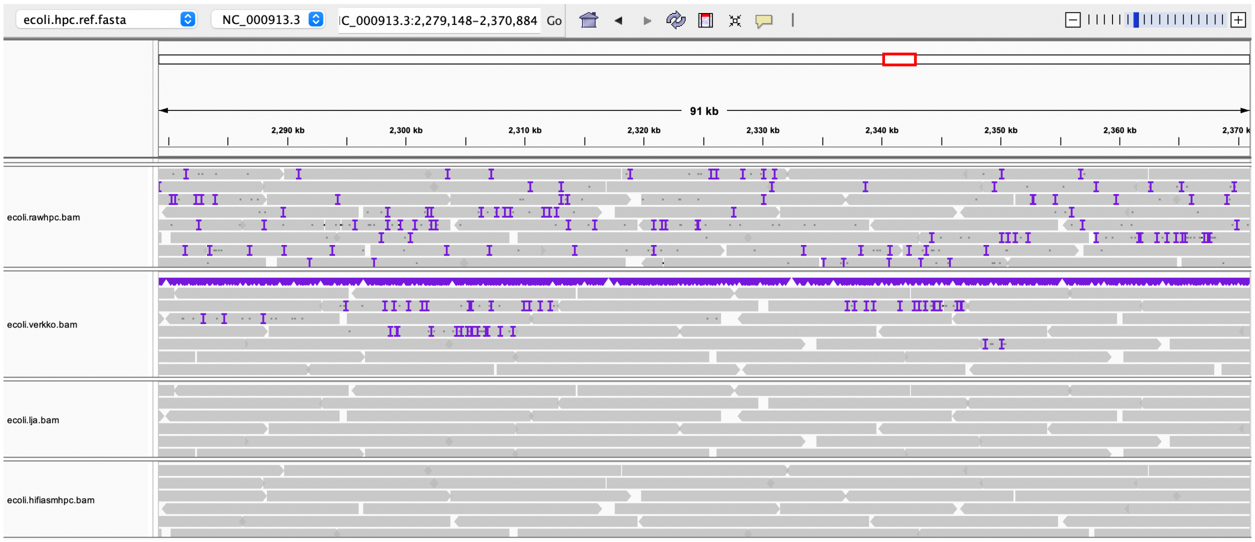

Supplementary Figure 2: IGV screenshot of OCs of *E. coli* reads by Verkko in a selected region across the whole genome in HPC space. The display window is set to be 91kb.

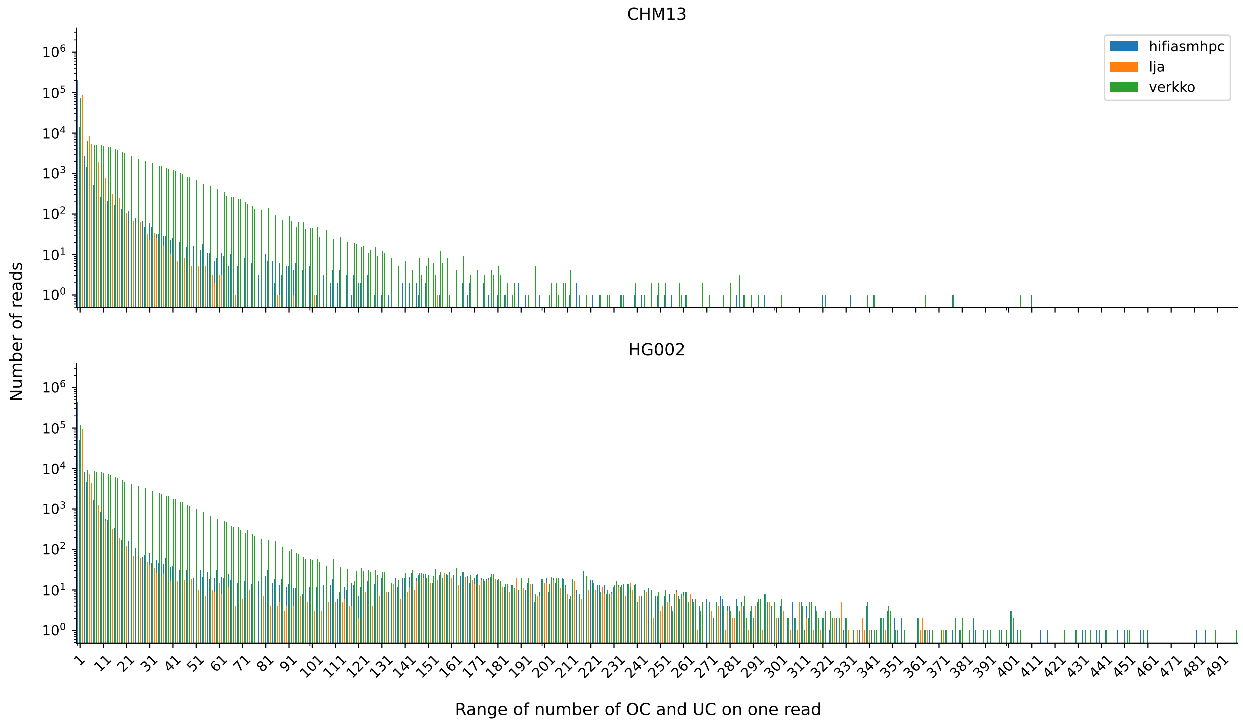

Supplementary Figure 3: Histogram of distribution of corrected reads by number of OC and UC on one read of the three tools. The number of correction errors ranges from 1 to 500. HPC raw reads is used for Hifiasm EC to keep the evaluation consistent.

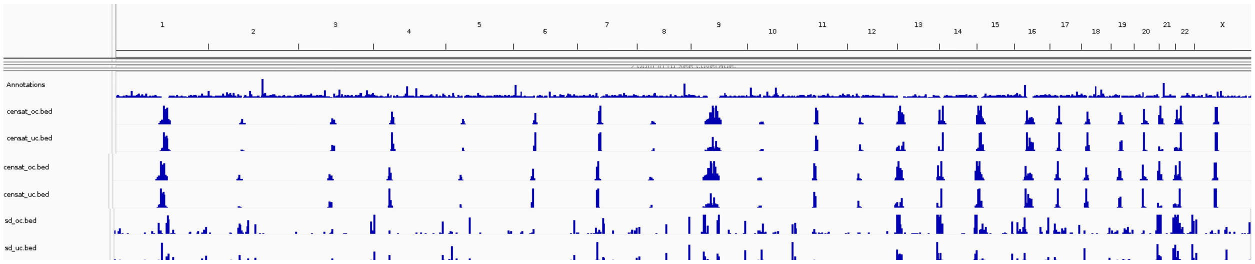

Supplementary Figure 4: IGV screenshot of OC and UC of CHM13 reads by hifiasm in assembly-challenging regions across the whole genome. The display window is set to be 50kb.

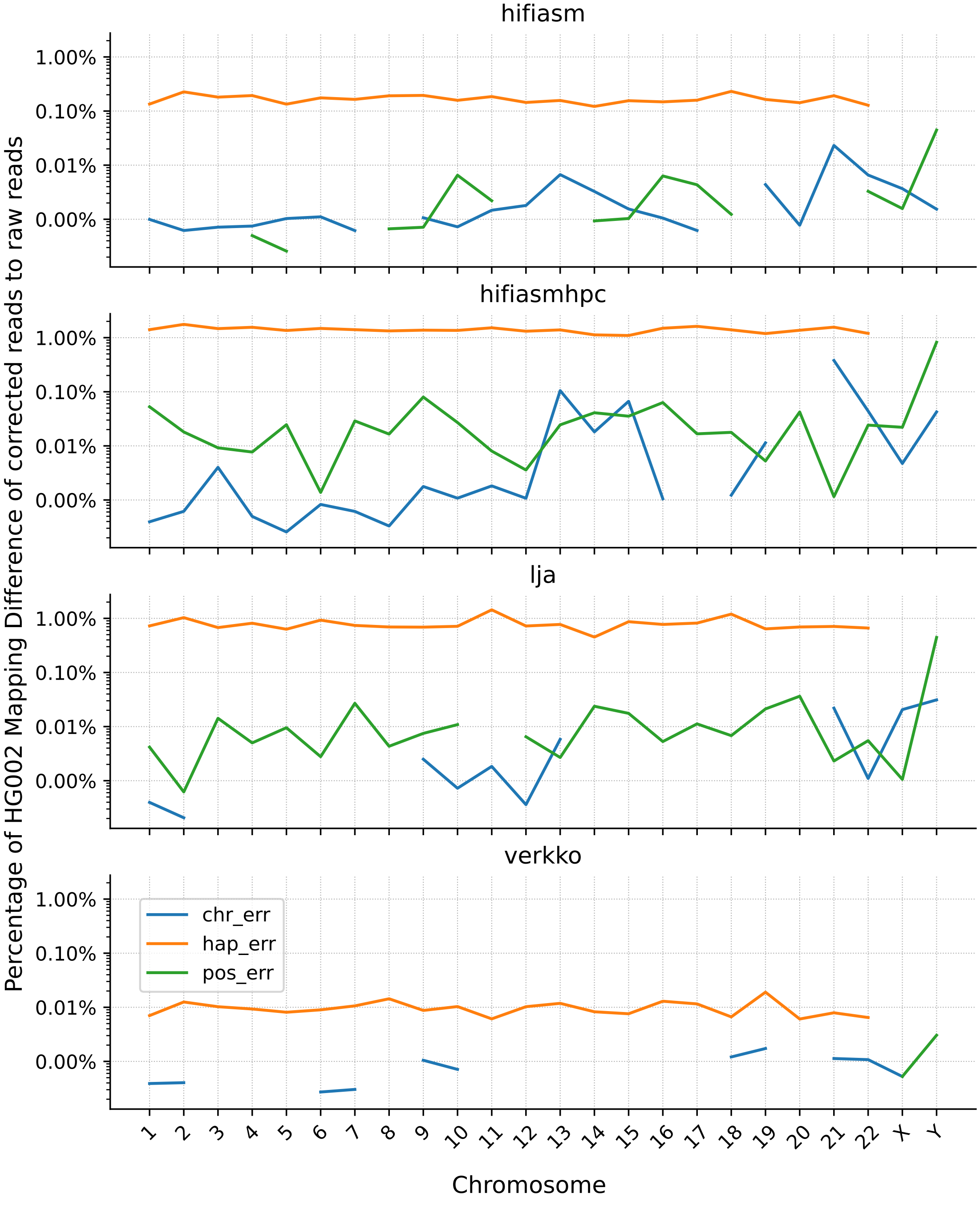

Supplementary Figure 5: Percentage of HG002 Mapping Difference of corrected reads to raw reads against Verkko-assembled reference for each chromosome.

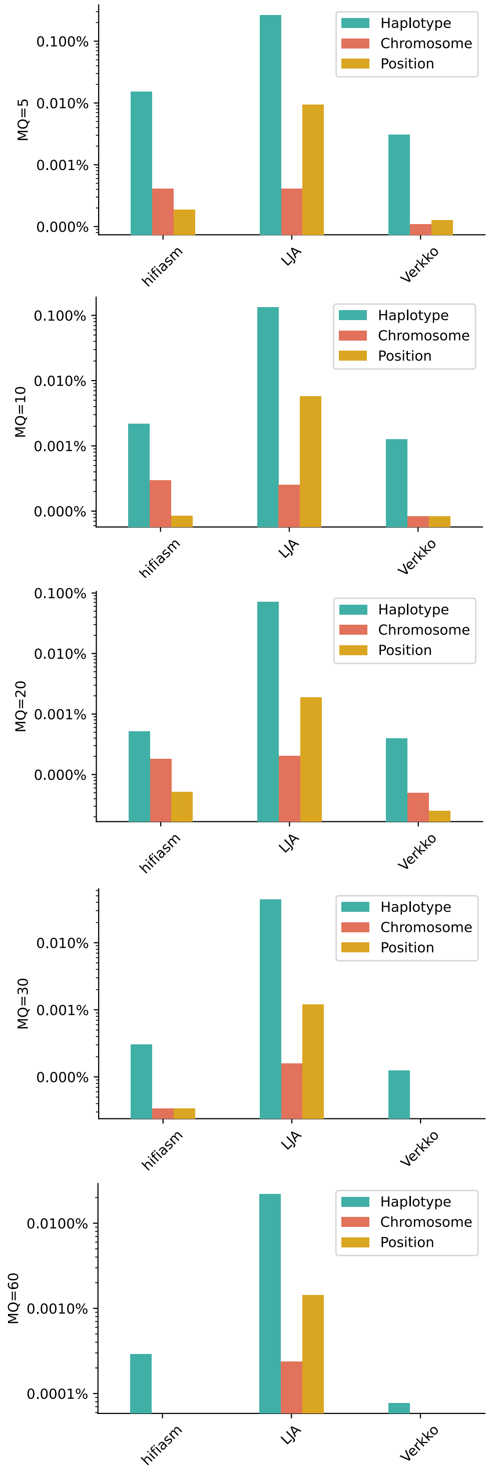

Supplementary Figure 6: Percentage of HG002 Mapping Difference of corrected reads to raw reads against Verkko-assembled reference. Reads alignments with mapping quality (MQ) lower than the labeled threshold were filtered out.

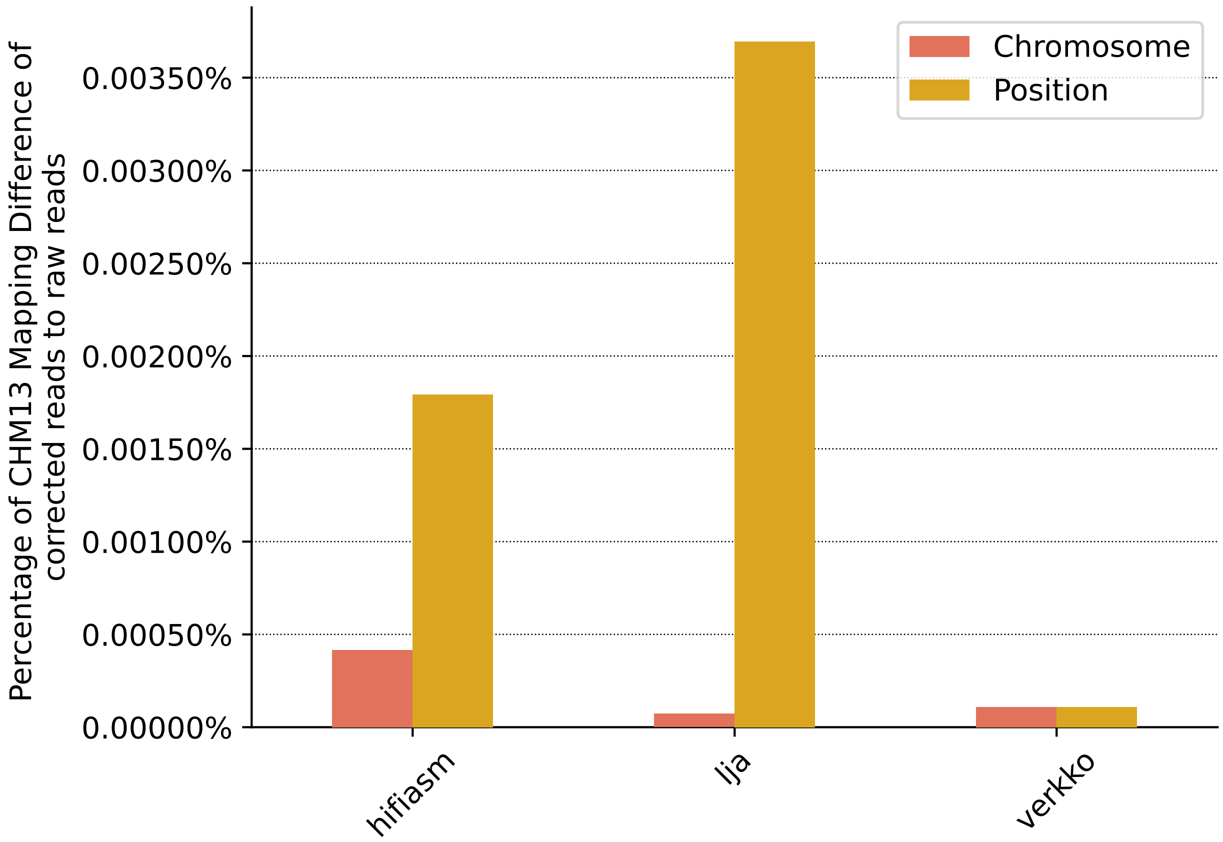

Supplementary Figure 7: Percentage of CHM13 Mapping Difference of corrected reads to raw reads. Chromosome and Position errors are included here

Mapping Difference between CHM13 raw reads and Hifiasm corrected reads against reference genome

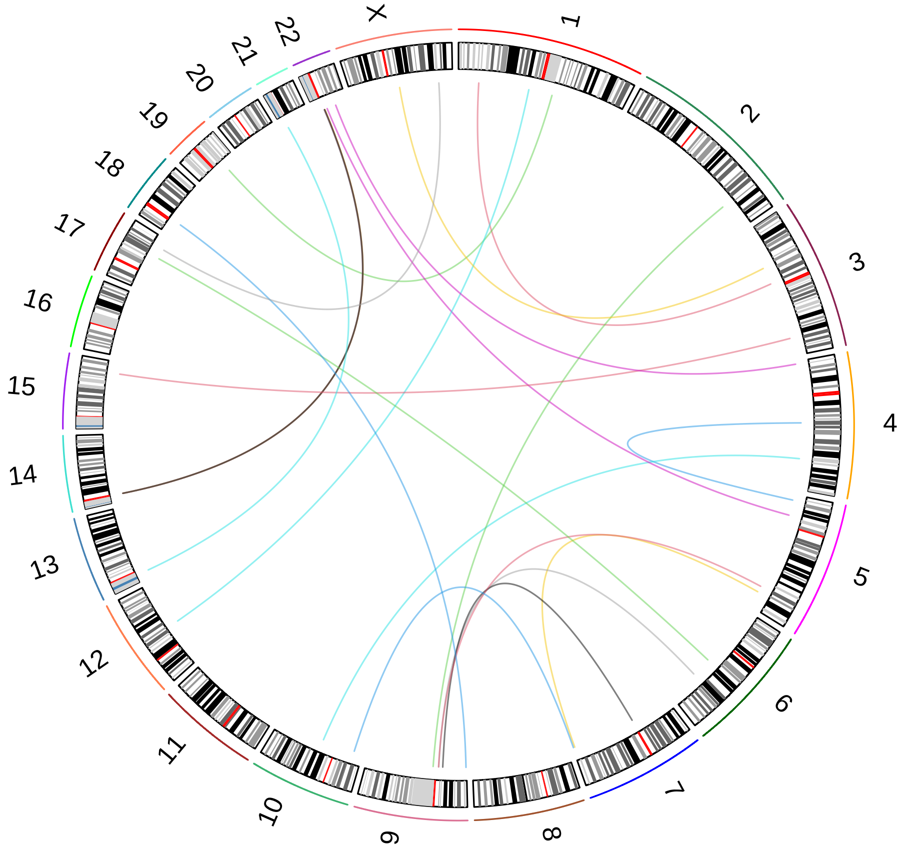

Supplementary Figure 8: Circos plot of Mapping Difference between CHM13 raw reads and Hifiasm corrected reads against reference genome (Mapping quality=60)

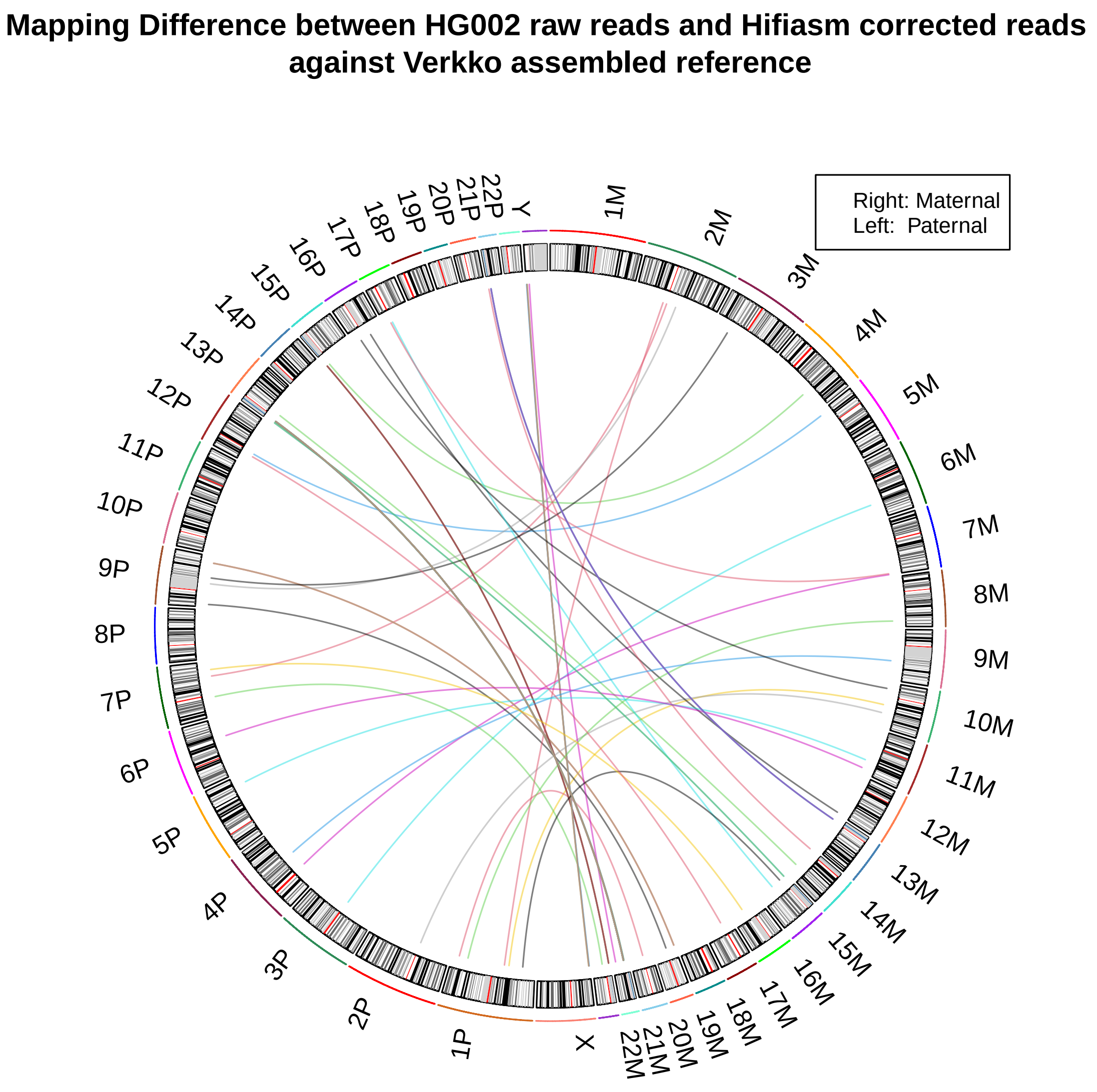

Supplementary Figure 9: Circos plot of Mapping Difference between HG002 raw reads and Hifiasm corrected reads against Verkko assembled reference (Mapping quality=60). Mapping ideogram is constructed using CHM13v2.0.

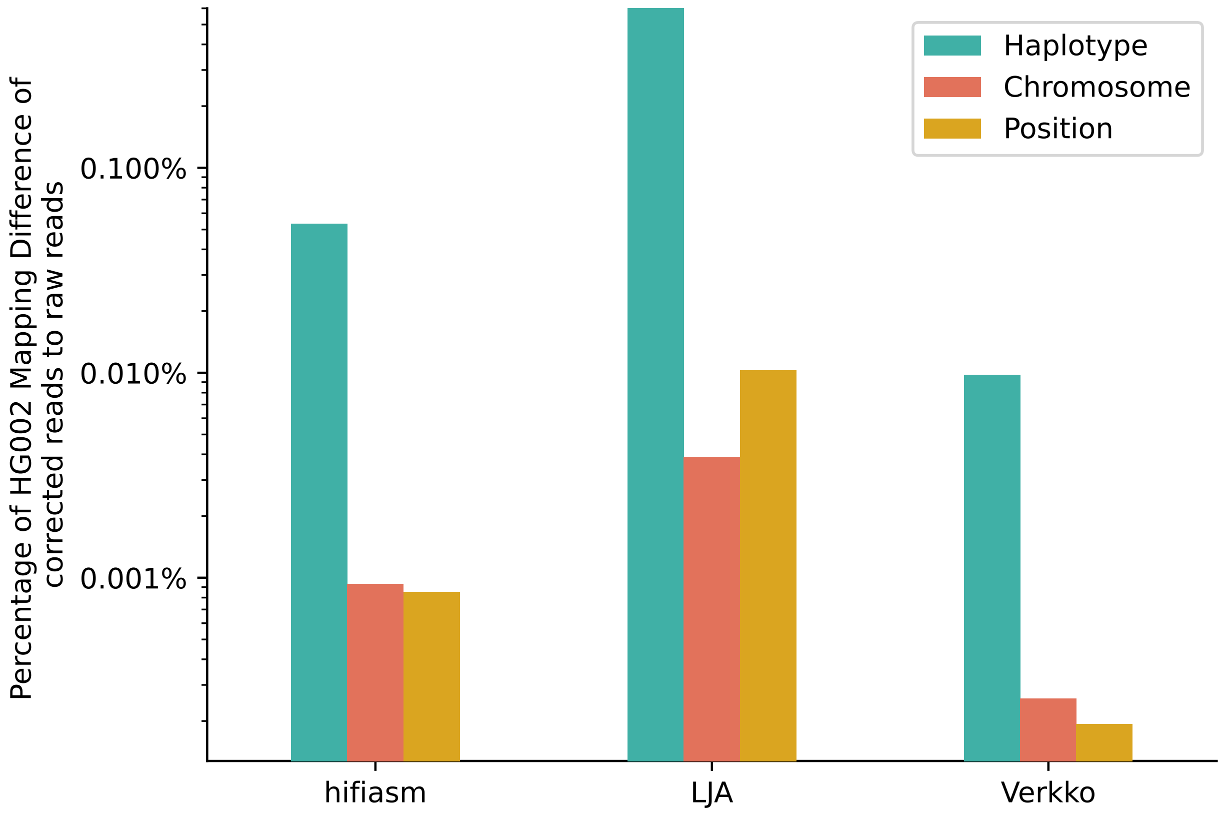

Supplementary Figure 10: Percentage of HG002 Mapping Difference of corrected reads to raw reads against Hifiasm-assembled reference. Haplotype and Position errors are included here.
